# Mr. Marshall on Vaccination

**Published:** 1830-04-01

**Authors:** 


### XXVII.

Popular Summary of Vaccination. By J. Marshall, Esq. 8vo. pp. 95. 1830.

Mr. Marshall's motto runs thus : " the increase of population is the increase of wealth." Certainly as far as the accoucheur, the vaccinator, and doctors of all kinds are concerned, there may be some truth in the motto ; but we much doubt the validity of Smith's dogma generally. Be that as it may, this slender volume has slender claims to notice among professional readers, for although we read it through, during a journey to Hampstead one morning, we were unable to discover any thing new, except the following extraordinary case.

" In August, 1829, a healthy sucking babe, six months old, was vaccinated, in fellowship with twelve other infants, from the vesicles of an eighth-day case ; the latter number went through the relative degrees of vaccination in the usual style. On the eighth day several of these were confronted with the phenomena of this unusual case. The mother stated that her child had an eruption, which came out the day before (seventh), and wished it to be examined. The patient had, from the effect of the operation, four vesicles on the right arm and three on the left, correct as to form and size, with a pearly hue, but the areola forwarder than is usual on the eighth day, exceeding an inch in diameter. The eruption appeared on the face, extending over the body, but thinly scattered, amounting to about fifty, mostly two or three inches asunder. Each eruptive pock bore a beautiful miniature resemblance, about half the size, of those on the arms effected by the operation ; the diameter of each eruptive vesicle exceeded the eighth of an inch, circular, indented in the centre, and elevated edge, the surface of a polished pearly lustre, and surrounded by a rose-coloured areola, half an inch in diameter.

" This eruption may be deemed to be *sui generis*, differing from all others, and by no means partaking of the varioliform type. The case is so extremely rare that no one, it is surmised, has met with its fellow : it yields an exception to a general rule, a departure from the usual law of the disease and the animal economy, in nosology, from its rarity, incapable of classification, and it can only be viewed as a capricious play of nature."

Mr. Marshall strongly recommends at least three punctures in each arm, and informs us that, " in almost every part of the united Empire, either a solitary vesicle, or only one in each arm, is formed." If this be the case, it is high time that country-practitioners should alter their practice. We wonder where they procure their lymph, if they confine themselves to such paucity of vesicles ! Re-vaccination is advised by Mr. Marshall. The extensive field which our author has had, as a public vaccinator, must have afforded him some novel materials, if such existed ; but we imagine the subject of vaccination is now completely exhausted, and that, if a Mosely were to rise from the grave, he would not be able to invest it with a single new attribute.

